# Comorbidity of mental disorders in synthetic cannabinoids abuse: clinical dynamics, behavior, adaptation

**DOI:** 10.1192/j.eurpsy.2024.991

**Published:** 2024-08-27

**Authors:** G. Selivanov, N. Bokhan

**Affiliations:** ^1^Mental Health Research Institute, Tomsk; ^2^ Saint Petersburg University of State Fire Service of Emercom of Russia; ^3^ Psychiatric Hospital of St. Nicholas the Wonderworker; ^4^Rehabilitation Treatment Center “Child Psychiatry, St. Petersburg; ^5^Siberian State Medical University, Tomsk, Russian Federation

## Abstract

**Introduction:**

The study of the phenomenon of deformation of mental disorders, clinical dynamics, behaviors, and adaptations in case of abuse of synthetic cannabinoids is of relevance.

**Objectives:**

To study the phenomenon of deformation of mental disorders, clinical dynamics, behaviors, and adaptations in case of abuse of synthetic cannabinoids

**Methods:**

Catamnestic, clinical-psychopathological methods (PANS, SANS, CGI, MMPI, CGI, STAI, LSI, TPA, ICD-10), statistical (Python 3.11.0).

**Results:**

291 men (age from 18 to 35 years) were examined: 240 - F12.2xx, of which 98 - F60.xx-F62.xx, 142 - F20.xx and 51 - F20.xx without substance abuse. The study took place from 2018 to 2023 based on psychiatric institutions of the Russia, Tomsk region, St. Petersburg, Noyabrsk and Nizhnevartovsk.

**Conclusions:**

The phenomenon of abuse of synthetic cannabinoids is a factor in the deformation of mental disorders. Persistent exogenous visual and delusional disorders contribute to the symptoms of exacerbations of schizophrenia; schizophrenic symptoms are included in psychotic episodes in personality disorders. In remission of schizophrenia, there is a quasi-adaptation from socio-professional environments, mostly addictive and criminalized, a pronounced smoothing of emotional impoverishment, a stigmatizing symptom is mainly a volitional defect, as well as frequent rehospitalizations not indirectly related to drugs. In remissions of drug use in patients with personality disorder, persistent schizophrenia of behavior. In patients with schizophrenia and patients with personality disorders, there is a distortion of behavior with a predominance in patterns of inclinations to delict, nonconformity, isolation in an addictive environment, suspiciousness. Drug abuse may initiate auto-aggression predominantly in individuals with personality disorders and to a lesser extent in patients with schizophrenia. True suicides in drug users include only episodes with depressive symptoms.

**Disclosure of Interest:**

None Declared

